# Pulsed ultrasound-stimulated microbubbles alter the tumor blood supply to enhance the efficacy of high-intensity focused ultrasound ablation of vascular tumors

**DOI:** 10.3389/fonc.2026.1708528

**Published:** 2026-01-30

**Authors:** Dong Luo, Min Yang, Jiajun Guo, Ying Hu, Le Liu, Yi Ren, Wenzhi Chen

**Affiliations:** 1 State Key Laboratory of Ultrasound in Medicine and Engineering, College of Biomedical Engineering, Chongqing Medical University, Chongqing, China; 2 Interventional Department, Chengdu Second People’s Hospital, Sichuan, China; 3 Department of Ultrasound, Guizhou Provincial People’s Hospital, Guizhou, China

**Keywords:** angiogenesis, high intensity focused ultrasound, microbubble, pulsed ultrasound, vascular tumor

## Abstract

**Objective:**

To investigate the therapeutic effect and mechanism of Pulsed ultrasound stimulated microbubbles to form thrombus and enhance the effect of high-intensity focused ultrasound (HIFU) for ablation of vascular tumors.

**Methods:**

After EOMA cells were inoculated subcutaneously in mice to form a hemangioendothelioma model, the mice were treated with HIFU alone, pulsed ultrasound combined with microbubbles therapy(PUCM) and the combination of the two methods(PUCM+HIFU) by Contrast ultrasound imaging, HE staining, CD31 immunofluorescence staining to detect the successful establishment of the model and the therapeutic effect. The effect of different treatment groups on apoptosis was detected by TUNEL staining. qRT-PCR, ELISA, CD31 and VEGF immunofluorescence staining were used to detect the changes of cytokines related to thrombosis and angiogenesis. The safety of the treatment was verified through HE staining of important organs and blood biochemical tests.

**Results:**

We successfully established hemangioendothelioma model, and the characteristics of CEUS are consistent with clinical findings. Pulsed ultrasonic stimulation of microbubbles can form thrombus. Further, PUCM+HIFU exhibits safety and significantly higher ablation effect and promotes apoptosis and necrosis of tumor cells. In addition, PUCM+HIFU can effectively lower the secretion of cytokines related to angiogenesis.

**Conclusion:**

In this study, a new therapeutic approach for vascular tumors was proposed, pulsed ultrasound stimulated microbubbles to form thrombus in vascular tumors and then combined with high-intensity focused ultrasound to treat vascular tumors. The combined therapy can better ablate vascular tumors and reduce the formation of blood vessels in the tumors. It is expected to provide a new scheme for the clinical treatment of vascular tumors.

## 1 Introduction

Vascular diseases can be divided into two main categories, vascular tumors and vascular malformations. The International Society for the Study of Vascular Abnormalities (ISSVA) defines vascular abnormalities with abnormal endothelial cell proliferation as vascular tumors (1), which contain multiple subtypes, but their uniform pathological features are abnormal endothelial cell (EC) proliferation and endothelial tissue structure disorder.

Infantile hemangioma is the most common benign vascular tumor, with an incidence rate of approximately 4% to 5% (2). In 90% of cases, it will completely disappear by the age of 4 (3, 4). In addition to the common infantile hemangiomas, there are also hemangioendothelioma, which is more common in young adults and is predominantly male. The histological manifestations of hemangioendothelioma are diverse and can be classified as epithelioid type, spindle cell type, reticular type, and Kaposi’s type, etc. (5, 6). Among them, epithelioid hemangioendothelioma is the most common subtype, characterized by tumor cells arranged in an epithelial-like pattern and often accompanied by lymphocyte infiltration. Hemangioendothelioma, which is usually located in the superficial or deep soft tissues of the extremities, may also involve bone (7). In about two-thirds of patients, the disease is multifocal, often involving multiple tissue planes.

Hemangioendothelioma is a type of tumor that originates from vascular endothelial cells. Its biological behavior lies between benign hemangioma and malignant angiosarcoma, and its clinical manifestations and prognosis show high heterogeneity (8). In recent years, the development of molecular pathology has greatly advanced our understanding of the pathogenesis of hemangioendothelioma. Specific gene rearrangements and dysregulation of signaling pathways are considered to be the key factors driving tumor occurrence and development. In epithelioid hemangioendothelioma (EHE), the WWTR1-CAMTA1 fusion is the most common characteristic genetic alteration (9–11). The abnormal protein encoded by this fusion gene can activate the downstream effectors of the Hippo signaling pathway, thereby promoting cell proliferation and survival (12). Clinically, EHE carrying this fusion usually presents with a relatively chronic course (13). The YAP1-TFE3 fusion is another important but relatively rare type of fusion in EHE (14). Compared with the WWTR1-CAMTA1 fusion type, the YAP1-TFE3 fusion type EHE shows stronger invasiveness and a poorer prognosis (15). Besides gene fusion, the abnormal activation of multiple key signaling pathways is also a key cause of the development of vascular endothelioma. The vascular endothelial growth factor (VEGF) pathway is a key pathway regulating angiogenesis. In various vascular tumors, the VEGF signaling pathway is abnormally activated, promoting the proliferation of endothelial cells, migration, and the formation of new blood vessels, thereby providing nutrients for tumor growth (16–18). The RAS/MEK/ERK pathway, as a classic proliferative signaling pathway, the abnormal activation of this pathway is also seen in some vascular tumors, usually related to the activation of upstream receptor tyrosine kinases or RAS gene mutations (19).

Due to the rarity and heterogeneity of hemangioendothelioma, there is currently a lack of standardized treatment guidelines. The formulation of treatment plans requires comprehensive consideration of the tumor subtype, location, extent, patient age, and overall health status. For localized, completely resectable lesions, surgical treatment is the preferred and potentially curative method (20). However, surgical resection is invasive, and patients have poor compliance, making it applicable only to localized and resectable lesions. Radiotherapy can be used for lesions that cannot be completely resected by surgery, as an adjuvant treatment after surgery to reduce the risk of local recurrence, or as a palliative treatment for patients who are not suitable for surgery (21, 22). However, radiotherapy can also cause some serious side effects, such as radiation dermatitis or ulcers, changes in skin pigmentation, cataracts, and carcinogenesis (23). Traditional chemotherapy drugs such as docetaxel and paclitaxel still have some efficacy in some patients, but the response rate and duration are limited, and they usually come with significant toxic side effects (20, 24, 25). Therefore, we urgently need a safe, effective, non-invasive, and side-effect-minimal treatment method.

As a safe, non-invasive and novel treatment method that can protect tissue function, focused ultrasound has been used in clinical treatment of various solid malignant tumors, including solid malignant tumors in pancreas, liver, kidney, bone, prostate and breast, as well as uterine fibroids and soft tissue sarcomas (26). Focused ultrasound therapy has characteristics of safety, effectiveness, short treatment time, intact skin and mucosa, and quick postoperative recovery (27). However, when using HIUF to treat tumors with abundant blood supply, the abundant blood flow leads to heat loss (28–30); thus, energy is difficult to be stored, and the ablation effect is poor. According to the existing research and reports, we have found that pulsed ultrasound combined with microbubbles may effectively solve this problem. According to the research conducted by Yang et al, microbubbles can be used to enhance the cavitation effect of low-frequency ultrasound and can target the destruction of the tumor’s new blood vessels. Microbubbles can lower the threshold of the ultrasonic cavitation effect, enhance the cavitation effect, destroy the vessel wall, activate endogenous or exogenous coagulation, and induce large-area capillary embolism (31). According to the report by Shen et al, the energy generated by the cavitation effect not only increases the permeability of the cell membrane, but also effectively destroys microvessels and expands the gap between endothelial cells. At the same time, they will mediate the destruction of the vascular endothelial layer, expose the subendothelial layer of the vessel, cause vascular thrombosis, and block the blood supply of malignant tumor tissues (32).

Therefore, we hereby hope to explore whether the combination of pulsed ultrasound and microbubbles for treating vascular tumors, followed by HIFU, can effectively enhance the ablation effect of the tumors. By stimulating microbubbles with pulsed ultrasound to reduce the abundant blood supply within the vascular tumors and form thrombus, it avoids the loss of high-intensity focused ultrasound energy due to the rich blood supply within the vascular tumors, thereby improving the ablation effect of focused ultrasound. Due to the difficulty in obtaining clinical samples, we established a reliable and commonly used animal model of hemangioendothelioma (33), and explored the therapeutic effect of high-intensity focused ultrasound on hemangioendothelioma and its potential mechanism after the combined action of microbubbles and pulsed ultrasound.

## 2 Materials and methods

### 2.1 Antibodies and reagents

The CD31 and VEGF antibodies were purchased from Proteintech Co., Ltd. FITC AffiniPure Goat Anti-Rabbit IgG (H+L) and DyLight 649 AffiniPure Goat Anti-Mouse IgG (H+L) were purchased from Earth Ox, USA. Hematoxylin & Eosin Dyeing Solution (H&E) purchased from Autogenic Bioengineering Shanghai Co., Ltd. The TUNEL FITC Apoptosis Detection Kit and anti-fluorescence quenching sealing tablets were purchased from Beyotime Biotechnology Co., Ltd. The ELISA kits for D2D, FI, FIII, FXII, VEGF, and VEGFR-2 were purchased from Jingmei Biotechnology (Jiangsu, China). RNA isolater Total RNA Extraction Reagent, HiScript III RT SuperMix for qPCR (+gDNA wiper) and ChamQ SYBR qPCR Master Mix were purchased from Vazyme Biotech Co.,Ltd. SonoVue (Sulphur Hexafluoride Microbubbles) was purchased from Bracco Co., Ltd.

### 2.2 Cells and animals

Mouse hemangioendothelioma cells (EOMA) were purchased from Shanghai Fuheng Biotechnology Co., Ltd.

BALB/c nude female mice weighing about 15-20g and 4–6 weeks old were provided by the Changzhou Kavins Animal Laboratory animal Co., LTD. All animal experiments were conducted in accordance with the guidelines of the Laboratory Animal Center of Chongqing Medical University and approved by the Ethics Committee of Chongqing Medical University (IACUC-CQMU-2024-0830). All animal experiments were also complied with the Guide for the Care and Use of Laboratory Animals and the Animal Research: Reporting of *In Vivo* Experiments (ARRIVE)guidelines. After the treatment, mice were euthanized with pentobarbital sodium (40mg/kg), and the tumor size was measured in two dimensions to generate a tumor volume using the formula: 0.5 x (length x width^2^).

### 2.3 Cells culture

The EOMA cells were cultured with DMEM high-glucose medium containing 10% fetal bovine serum and 1% streptomycin - penicillin in a constant temperature of 37°C and an atmospheric oxygen environment of 5% CO2.

### 2.4 Establishment and grouping of animal models

Based on previous reports, we used EOMA cells to establish a subcutaneous hemangioendothelioma model. In short, mouse EOMA cells were incubated to a logarithmic growth phase and centrifugally collected, and the cell blast suspensions were re-suspended with sterile saline (PH = 7.4). EOMA cells with a density of 1×10^6^/100 μL were injected into the right subcutaneous area of the mouse. Mouse body weight and tumor size were measured 7 days after inoculation, and the experiment was performed when the tumor size of all tumor-bearing mice reached 300mm^3^. We euthanized mice and performed hematoxylin-eosin (HE), immunofluorescence (IF) staining to reveal the highly vascular structure of hemangioendothelioma. In short, hemangioendothelioma was fixed with 4% paraformaldehyde, embedded in paraffin blocks, and cut into 5 μm slices. The slices were incubated with CD31 primary antibody at 4 °C overnight. Use DyLight 649 AffiniPure Goat Anti-Mouse IgG (H+L) second antibody. The nuclei were re-stained with 4 ‘, 6-diaminidine 2-phenylindole (DAPI) and the images were captured under a confocal laser microscope. Imaging findings of hemangioendothelioma in mice were observed by contrastus-enhanced ultrasound (CEUS). Microbubble was injected into tail vein and real-time images were collected by ultrasound.

The 25 mice successfully modeled were randomly divided into 5 groups with the following treatments (n = 5): control group, pulsed ultrasound combined with microbubbles group (PUCM), high-intensity focused ultrasound group (HIFU), high-intensity focused ultrasound combined with pulse ultrasound and microbubbles group (PUCM+HIFU), and high-intensity focused ultrasound combined with pulse ultrasound and microbubbles group 7 days after the end of treatment(7DPUCM+HIFU).

### 2.5 Ultrasound therapy

#### 2.5.1 Pulsed ultrasound combined with microbubbles therapy

After successful modeling, mice were anesthetized by intraperitoneal injection of 0.3% sodium pentobarbital (50mg/kg). The mice were placed on the ultrasound treatment table immediately after 200 μL microbubble was injected into the tail vein. The tumor surface was treated with an ultrasonic coupler and then irradiated with a Focused Ultrasound Tumor Therapeutic System (Model-JC200, Chongqing Haifu Medical Technology Co., Ltd, Chongqing, China)

The parameters adopted in this study are as follows: PUCM: Operating frequency: 1 MHz, power: 20 W, duty cycle: 5%, repetition frequency: 1 Hz, operating time: 60 s, actual effective time: 3 s. (During this process, the cavitation effect generated by the pulsed ultrasound we mainly used damaged the vascular endothelial cells within the tumor, causing thrombosis).

#### 2.5.2 High intensity focused ultrasound therapy

After successful modeling, mice were anesthetized by intraperitoneal injection of 0.3% sodium pentobarbital (50mg/kg). The tumor surface was treated with an ultrasonic coupler and then irradiated with a Focused Ultrasound Tumor Therapeutic System (Model-JC200, Chongqing Haifu Medical Technology Co., Ltd, Chongqing, China).

The parameters adopted in this study are as follows: HIFU: Working frequency: 1 MHz, power: 120 W, working time: 1 second, actual effective time: 1 second. (During this process, we mainly utilize the thermal effect of continuous high-intensity focused ultrasound to ablate the vascular tumor after thrombosis).

High intensity focused ultrasound combined with pulsed ultrasound and microbubbles therapy (PUCM+HIFU)

One hour after the end of pulse ultrasound combined with microbubbles therapy (same as above) followed by high-intensity focused ultrasound (same as above).

### 2.6 Histopathological analysis

After the mice received different treatments, tumor tissues of the control group and the experimental group were collected, fixed with 4% paraformaldehyde solution, dehydrated, embedded in paraffin, sliced, stained with hematoxylin-eosin, and observed under an optical microscope (Olympus Co., LTD., Tokyo, Japan).

### 2.7 Detection of apoptosis in tumor tissue

Tumor tissues of mice were collected after treatment, fixed with 4% paraformaldehyde solution, dehydrated, embedded in paraffin, and sliced. To examine the apoptosis, TdT-mediated dUTP nick end labeling (TUNEL) was used to stain tumor cells by immunofluorescence staining. According to the manufacturer’s instructions, the TUNEL apoptosis detection kit was used, and the nucleus was stained with DAPI. The fluorescence was observed by confocal laser microscopy, and the fluorescence intensity was quantitatively analyzed by ImageJ software.

### 2.8 VEGF and CD31 immunofluorescence staining of tumor tissue

The tumor tissues of mice in each group after different treatment methods were made into paraffin sections and then stained with vascular endothelial growth factor (VEGF) and platelet-endothelial cell adhesion molecule (CD31) by immunofluorescence. After routine dewaxing and rehydration, antigen repair and sealing, the corresponding anti-antibody was added and incubated overnight in a refrigerator at 4°C. After washing with PBS for 3 times, the second anti-antibody was added and incubated at 37°C for 1h and then washed with PBS again for 3 times. Dye with DAPI solution at room temperature for 10 min, wash with PBS for 3 times, then add anti-fluorescence attenuation sealant, and cover the cover glass. The fluorescence was observed by confocal laser microscopy, and the fluorescence intensity was quantitatively analyzed by ImageJ software.

### 2.9 Contrast-enhanced ultrasound

The imaging features of hemangioendothelioma were observed by contrast-enhanced ultrasound after different treatments. Mice were anesthetized by intraperitoneal injection of 0.3% pentobarbital sodium solution (50mg/kg), and real-time images were collected by ultrasound diagnostic system after Microbubbles were injected into tail vein. The red dashed box indicates the tumor area, the green box indicates the region of interest (ROI) that we have selected. The changes in blood supply within the tumor were observed through CEUS. Multiple frames of images were selected, and the gray values of the ROI were quantitatively analyzed using the ImageJ software.

### 2.10 ELISA quantification assay

ELISA kits (Jiangsu Jingmei Biotechnology Co., Ltd., China) were used in accordance with the manufacturer’s instruction. Mouse D2D ELISA Kit, Mouse FI ELISA Kit, Mouse FIII ELISA Kit, and Mouse FXII ELISA Kit, Mouse VEGF ELISA Kit, Mouse VEGFR-2 ELISA Kit were employed to measure the expression of cytokine in serum.

### 2.11 Quantitative real-time polymerase chain reaction

Total RNA was extracted from tumor tissues according to the instructions of the cell/tissue total RNA extraction kit, the concentration was determined, the RNA was reverse-transcribed into cDNA according to the instructions of the reverse transcription kit. PCR amplification was then performed under the following conditions: 95 °C for 30 s, followed by 40 cycles of 95 °C for 5 s and 60 °C for 10 s, and CT values were obtained. Using GAPDH as internal parameters, the mRNA expression of each gene was calculated by 2-^ΔΔ^ CT method.

### 2.12 Safety assessment

After the treatment of the mice was completed, their important organs such as heart, liver, spleen, lung, kidney, brain, and skin were collected and processed into paraffin tissue sections for hematoxylin-eosin (H&E) staining. At the same time, after different treatment methods were completed, the mice’s eyeballs were removed and blood was collected. The blood was separated at a speed of 3000 rpm to obtain serum. The levels of serum biochemical indicators such as alanine aminotransferase (ALT), aspartate aminotransferase (AST), blood urea nitrogen (BUN), creatinine (CREA), creatine kinase (CK), and total bilirubin (TBIL) of each mouse were detected to evaluate the safety of the treatment in the body.

### 2.13 Statistical analysis

Statistical analysis was performed using GraphPad Prism version 9.0 for Windows (GraphPad Software; La Jolla, CA, USA). All data are expressed as mean ± standard deviation (SD) of at least three independent experiments. To determine significant differences among groups, a one-way ANOVA was performed, and a Student’s t-test was used to compare individual groups. A p-value of less than 0.05 was considered statistically significant. In the statistical chart, asterisks (*) denote p < 0.05, double asterisks (**) denote p < 0.01, triple asterisks (***) denote p < 0.001.

## 3 Results

### 3.1 Verification of successful establishment of animal models

**Figure 1:**
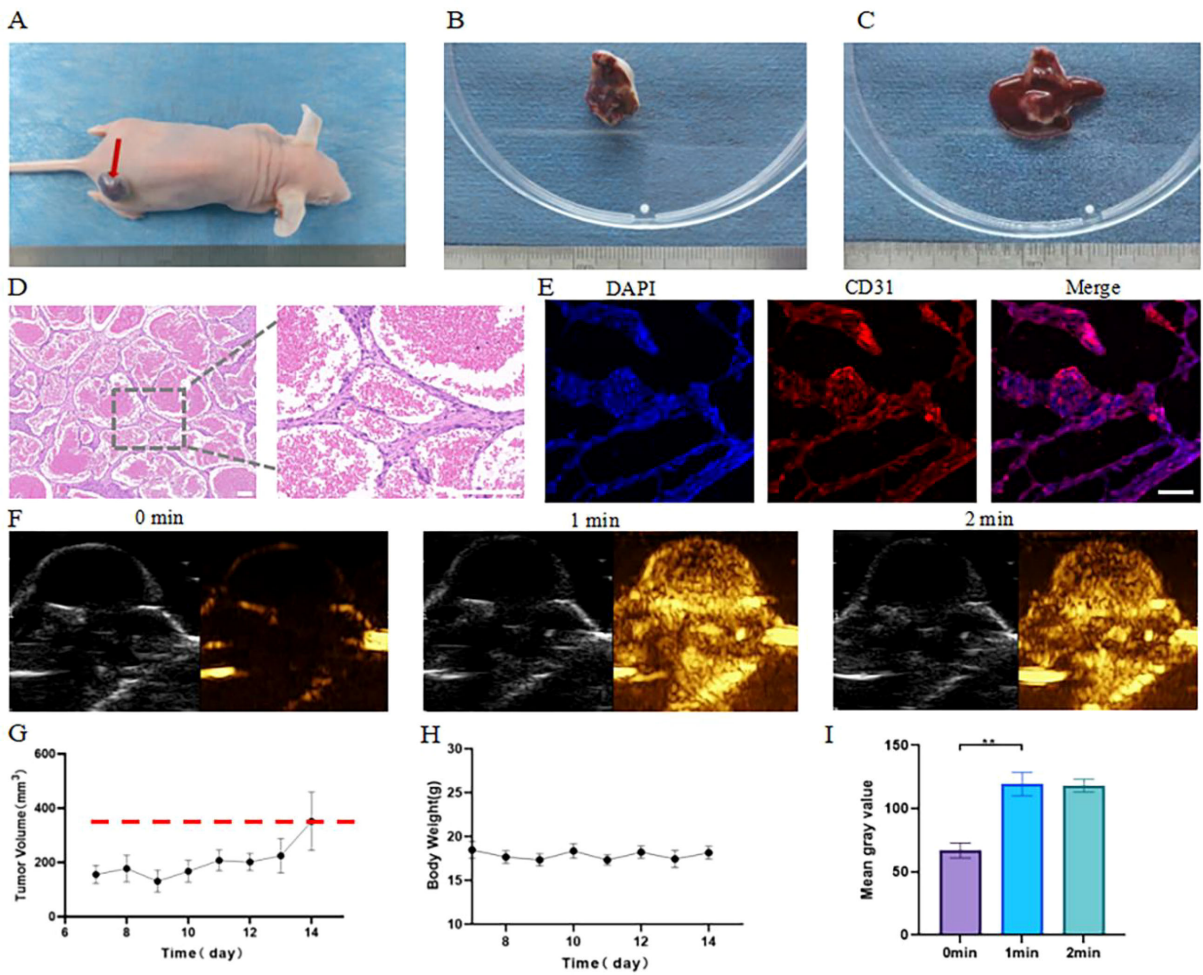
Establishment and verification of subcutaneous hemangioendothelioma model in mice. **(A)** Mice subcutaneous hemangioendothelioma; **(B)** Isolated mice hemangioendothelioma; **(C)** After incision of isolated mice hemangioendothelioma; **(D)** HE pathological section of hemangioendothelioma. Scale bars indicate 200 μm; **(E)** The expression of endothelial cells in hemangioendothelioma was observed by CD31 fluorescence staining. Scale bars indicate 100 μm; **(F)** Contrast-enhanced ultrasound images at different time points; **(G)** Changes of tumor volume after EOMA cell inoculation 7 days; **(H)** Changes of weight after EOMA cell inoculation 7 days; **(I)** Quantitative analysis of contrast-enhanced ultrasound mean gray value at different time points. **p < 0.01.

We successfully established mice hemangioendothelioma by subcutaneous injection of EOMA cells (**Figure 1A**). One week after cell injection, the tumor began to grow. During this period, the weight of the mice showed no significant change (**Figure 1H**). One week later, the tumor volume reached 300 mm3 (**Figure 1G**), which was an appropriate size for HIFU ablation treatment. After CEUS and tumor incision, it was observed that the tumor contained abundant blood supply (**Figures 1B, C**), and its imaging manifestations were fast forward and slow out, obvious nodular enhancement in the tumor was observed 1 minute after contrast-enhanced ultrasound. (**Figures 1F I**). CD31 staining and HE staining showed high vascular characteristic structure in the tumor (**Figures 1D, E**). All of the above results indicated that the established mouse hemangioendothelioma were significantly different from conventional solid tumors and highly similar to human vascular tumors.

### 3.2 Analysis of pathological changes and apoptosis of tumor tissue

**Figure 2:**
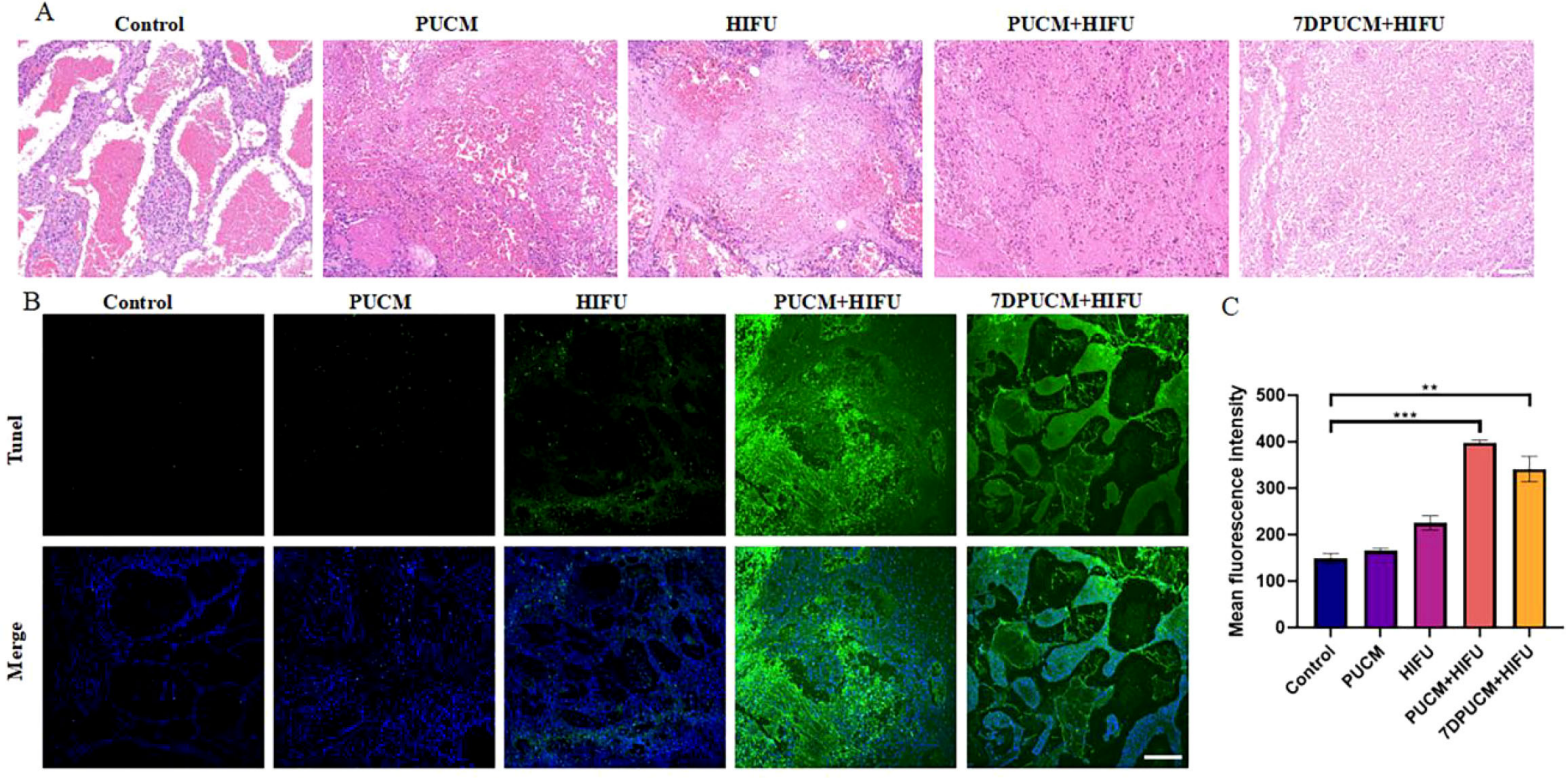
Histopathological analysis and cell apoptosis analysis after different treatments **(A)** HE pathological section of hemangioendothelioma after different treatments. Scale bars indicate 100 μm; **(B)** The expression of apoptosis in hemangioendothelioma was observed by Tunel fluorescence staining after different treatments. Scale bars indicate 200 μm; **(C)** Quantitative analysis of green mean fluorescence intensity after different treatments. **p < 0.01, ***p < 0.001.

The pathological changes of tumor tissues were observed by hematoxylin-eosin (H&E) staining, and the apoptosis of tumor tissues was observed by TUNEL immunofluorescence staining. H&E staining showed that, in the tumor area of the PUCM group, obvious thrombosis was observed and began to accumulate; in the HIFU group, a small number of tumor cells showed necrosis. Compared with other groups, tumor cells in the PUCM+HIFU group showed a large number of necrosis, cell structure was significantly changed, and nuclear fragmentation was increased (**Figure 2A**). Laser confocal microscopy showed that the green fluorescence intensity was weak in HIFU alone and in the combined microbubble group with pulsed ultrasound, and the green fluorescence was strongest in the combined HIFU group after pulsed ultrasound with microbubble, and the fluorescence intensity was also higher after 7 days of treatment (**Figure 2B**). Quantitative analysis by ImageJ software showed the strongest fluorescence signal in the combined treatment group compared with the other groups (**Figure 2C**), indicating increased apoptosis of tumor cells (p < 0.001). These results strongly confirm that combination therapy can better ablate the tumor.

### 3.3 Immunofluorescence detection of VEGF and CD31 expression after treatment

**Figure 3:**
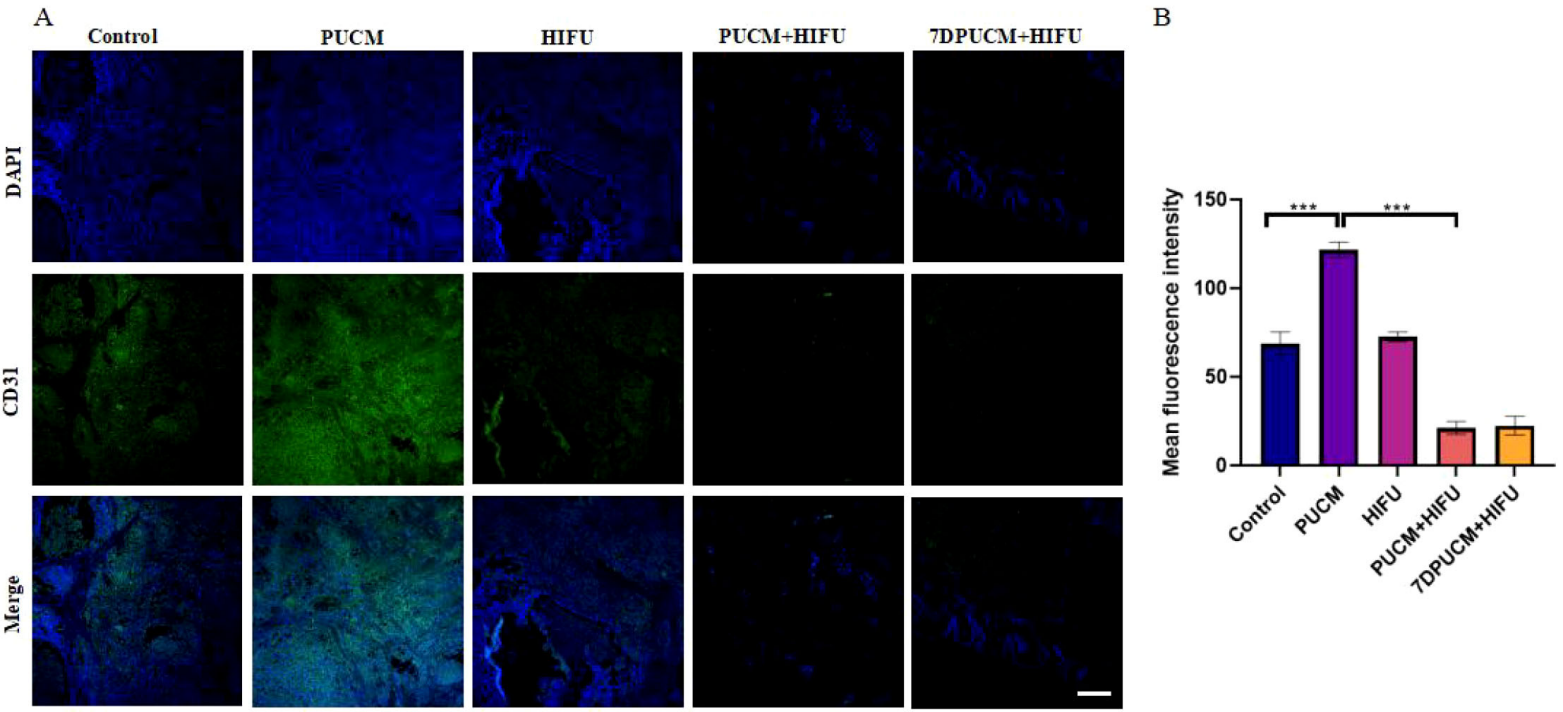
CD31 immunofluorescence staining after different treatments **(A)** The expression of CD31 in hemangioendothelioma was observed by CLSM after different treatments. Scale bars indicate 200 μm; **(B)** Quantitative analysis of green mean fluorescence intensity after different treatments. ***p < 0.001.

**Figure 4:**
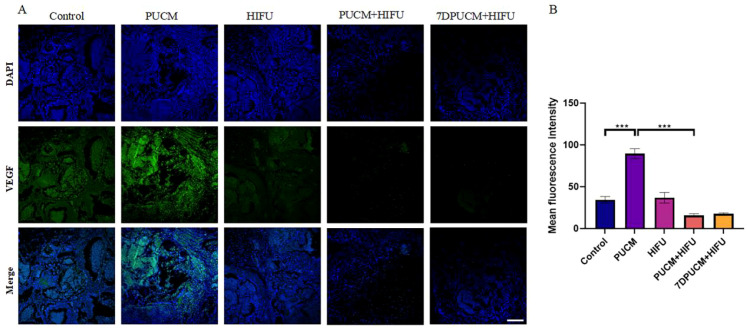
VEGF immunofluorescence staining after different treatments **(A)** The expression of VEGF in hemangioendothelioma was observed by CLSM after different treatments. Scale bars indicate 200 μm; **(B)** Quantitative analysis of green mean fluorescence intensity after different treatments. ***p < 0.001.

Immunofluorescence staining of VEGF and CD31 was performed on tumor tissues after different treatments to determine whether the combination therapy can effectively inhibit neovascularization in tumor tissues. The experimental results were shown in **Figures 3**, **4**. A large number of positive cells of VEGF and CD31 staining were expressed in tumor tissues of control group and non-combined treatment group. However, VEGF and CD31 positive cells in tumor tissue decreased significantly in combination treatment group and 7 days after combination treatment. Quantitative analysis by ImageJ software also showed that the PUCM+HIFU group had the weakest fluorescence signal (p < 0.001) compared with other groups, indicating that our combination therapy effectively inhibited angiogenesis and had a certain maintenance effect.

### 3.4 Analysis of contrast ultrasound imaging before and after treatment

**Figure 5:**
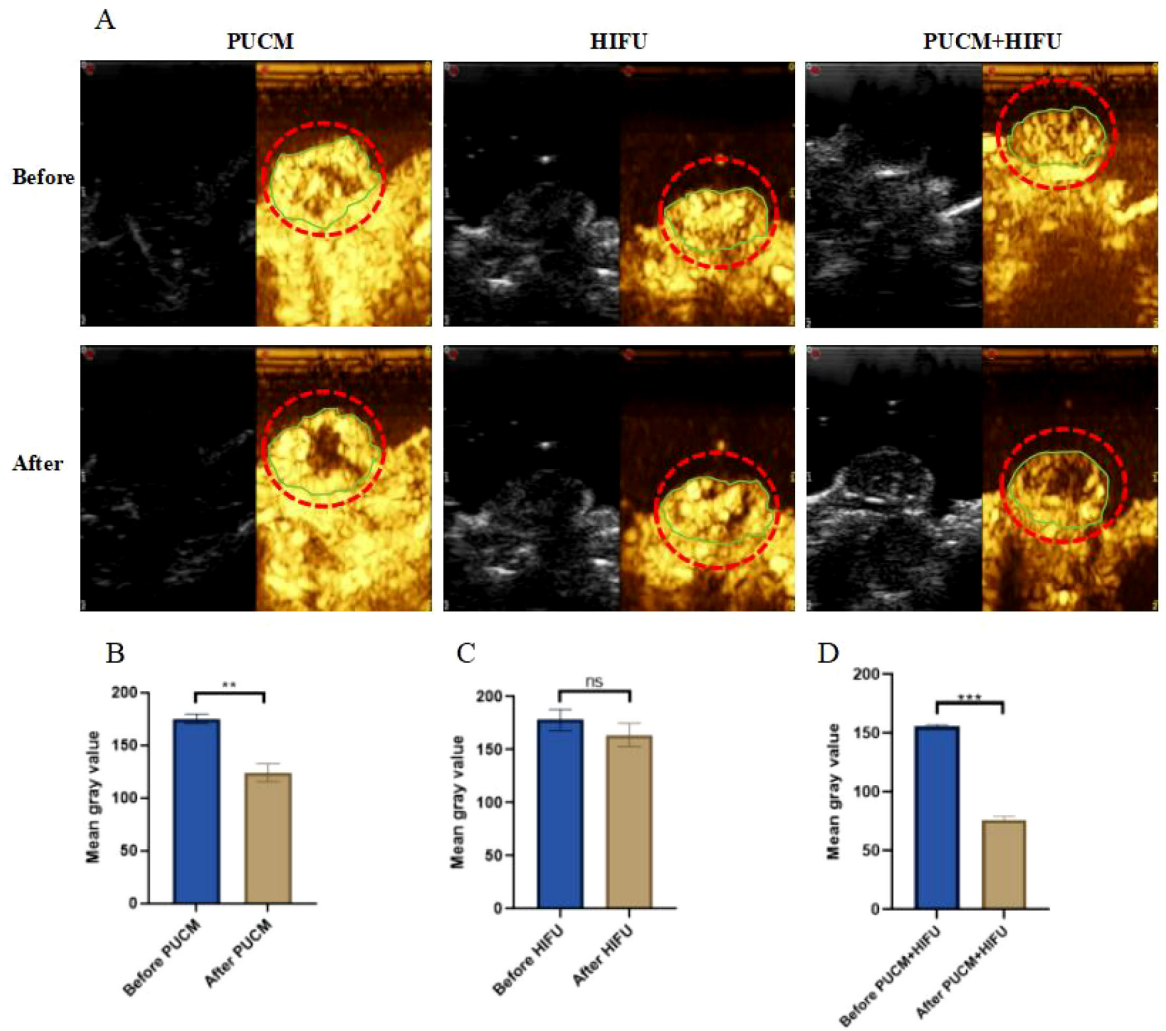
Contrast-enhanced ultrasound imaging before and after different treatments. **(A)** Contrast-enhanced ultrasound imaging; **(B–D)** Quantitative analysis of contrast-enhanced ultrasound mean gray value at different treatments. **p < 0.01, ***p < 0.001.

Contrast-enhanced ultrasound (CEUS) was used to study the changes of blood supply in the tumor before and after treatment and the therapeutic effect. In the pulsed ultrasound combined with microbubbles group, we could observe that the gray value in the tumor decreased significantly after treatment, and the reduction in ultrasound enhancement indicates that there have been hemodynamic changes within the tumor and thrombosis has occurred. In the HIFU group, the effect of ultrasonic enhancement is not obvious, indicating that the ablation effect of vascular tumors was poor and coagulation necrosis did not occur. In the combined treatment group, the effect of ultrasonic enhancement is more obvious than that in the single pulse ultrasound combined with microbubbles group, indicating that the combined treatment caused coagulation necrosis in the tumor and the ablation effect was good (**Figure 5**). The above results provided strong evidence for our speculation. By pulse ultrasound stimulate microbubbles to form thrombus within vascular tumors, the internal blood supply is reduced, avoiding the consumption of thermal energy by the HIFU due to the abundant blood supply within the vascular tumors, thereby improving the ablation effect of HIFU.

### 3.5 Detection of cytokines related to thrombosis and angiogenesis

**Figure 6:**
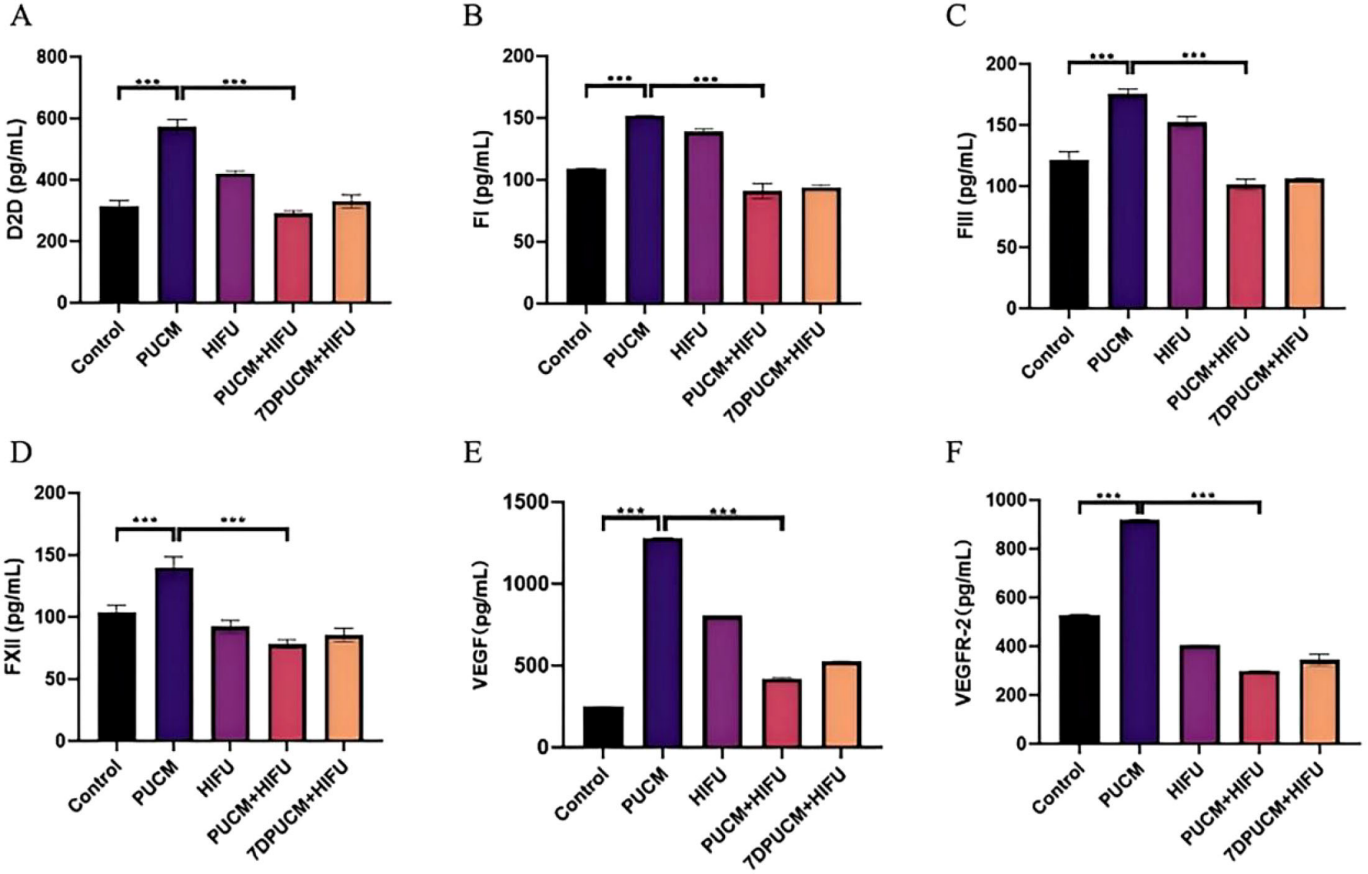
Results of the ELISA experiments. The levels of D2D **(A)**, FI **(B)**, FIII **(C)**, FXII **(D)**, VEGF **(E)**, VEGFR-2 **(F)** were measured by ELISA after the treatment of different experimental groups. ***p < 0.001.

The formation of thrombus and blood vessel is closely related to the secretion level of related cytokines. The cytokines D2D, FI, FIII and FXII associated with thrombosis and VEGF and VEGFR-2 associated with angiogenesis were detected by ELISA. Serum levels of D2D, FI, FIII and FXII in PUCM group were higher than those in other groups (p < 0.001), while serum levels of VEGF and VEGFR-2 in PUCM+HIFU group were lower than those in other groups (p < 0.001) (**Figure 6**).

### 3.6 Effect of different treatments on angiogenesis

**Figure 7:**
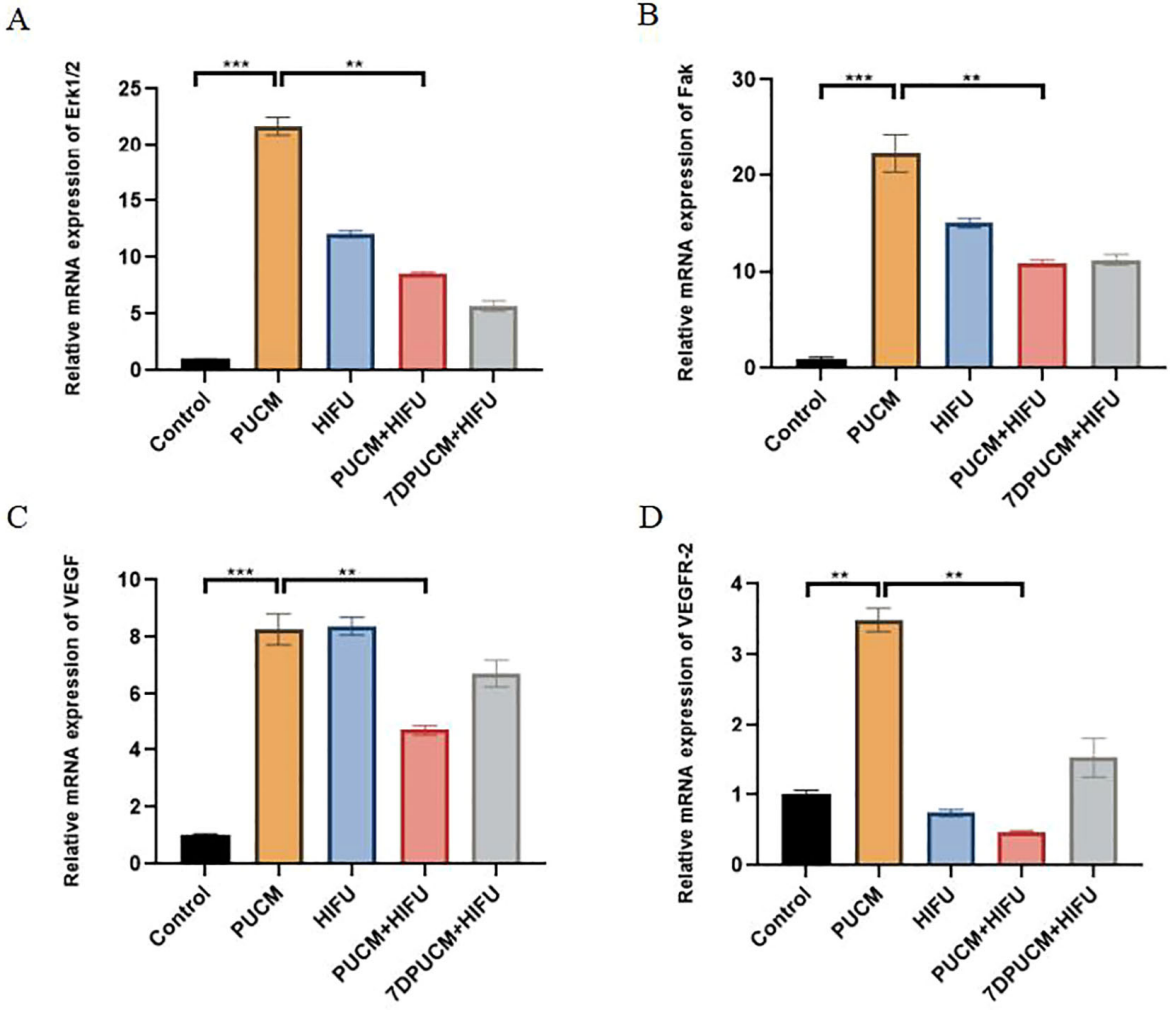
Results of the qRT-PCR experiments. The levels of Erk1/2 **(A)**, Fak **(B)**, VEGF **(C)**, VEGFR-2 **(D)** were measured by qRT-PCR after the treatment of different experimental groups. **p < 0.01,***p < 0.001.

The effect of different treatment methods on angiogenesis was further verified by qRT-PCR. As can be seen from **Figure 7**, the relative expression of genes mRNA related to angiogenesis was significantly decreased in the PUCM+HIFU group, which was significantly different from that in the other groups (P<0.01). It is speculated that combined therapy can not only improve the ablation effect of HIFU but also inhibit angiogenesis in tumors.

### 3.7 Safety assessment of different treatment methods

**Figure 8:**
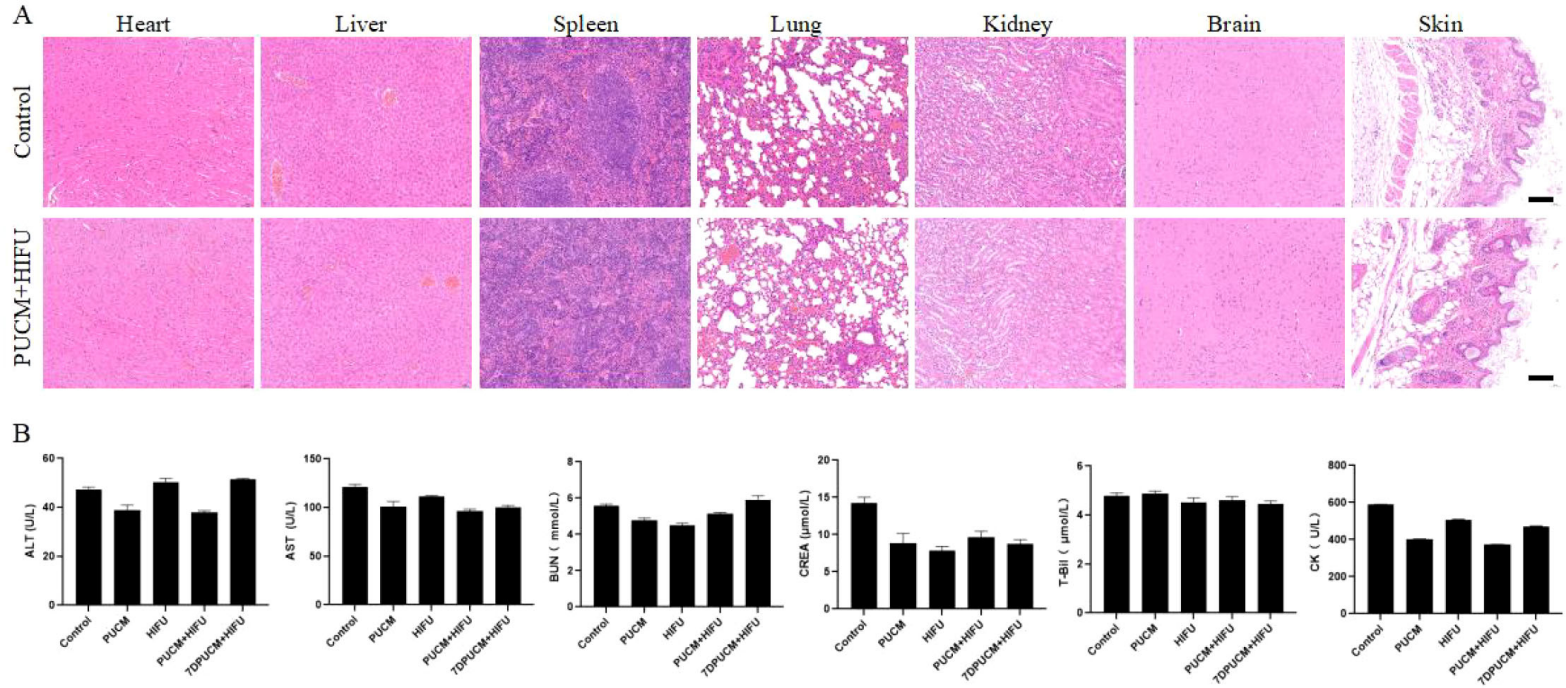
Results of the safety experiments. **(A)** HE images of important organs. Scale bars indicate 100 μm; **(B)** Results of liver, kidney, and heart function indicators after different treatments.

The safety of the combined treatment was evaluated by observing the histological sections of important organ tissues from the vascular endothelial tumor mice after combined treatment and by detecting their blood biochemistry. The H&E pathological tissue staining results (**Figure 8A**) showed that after the treatment, the major organs of the PUCM + HIFU group mice, such as the heart, liver, spleen, lungs, kidneys, brain, and skin, did not show obvious cellular structure and histological damage compared to the control group. This proved that the combined treatment did not cause significant damage to normal tissues.

The blood biochemistry test results are shown in **Figure 8B**. The liver function indicators (ALT and AST), kidney function indicators (BUN, CREA, and TBIL), and heart function indicators (CK) of the mice showed no significant differences compared to the control group, indicating that the treatment did not induce liver, kidney, and heart function damage in the mice. The above experimental results all prove that the combined treatment has good safety.

## 4 Discussion

As a non-invasive surgical treatment technique, focused ultrasound therapy has been widely used to treat benign and malignant solid tumors in various parts of the body in recent years and is currently a safe and effective means for the treatment of solid tumors (34–38). The principle of treatment is to use the physical characteristics of ultrasound to gather low-energy ultrasound *in vitro* at the target area *in vivo*, and to generate transient high-temperature effect, cavitation and mechanical effect through high-energy ultrasound at the target area, so that tumor tissue will immediately undergo coagulation necrosis (36, 37). Focused ultrasound can also destroy tumor small blood vessels and capillaries, cause tumor blood vessels to embolize (39), and induce cell apoptosis (40, 41). However, compared with tumor tissue and small blood vessels, large blood vessels seem to be less susceptible to HIFU damage, which may be due to the abundant blood flow in large blood vessels, which dissipates heat energy from the blood vessel wall (23). Due to the special structure of vascular tumors, the abundant blood flow inside them will lead to the thermal energy consumption generated by HIFU affecting the ablation effect. Conventional dose of HIFU cannot directly ablate vascular tumors. This study reports that in the treatment of vascular tumors, the use of pulsed ultrasound combined with microbubbles to form thrombus, and then combined with HIFU treatment, is expected to enhance the ablation effect of HIFU and inhibit the formation of tumor blood vessels.

In recent years, it is a new idea to combine HIFU with other therapeutic techniques to improve the therapeutic effect. For example, new microbubble materials such as nano-lipid fluorocarbon microbubbles are used to enhance the therapeutic effect of HIFU. This combined treatment can improve the acoustic characteristics of tumor tissues and promote drug delivery (42). Because clinical specimens of vascular tumors are difficult to obtain, we established and validated a commonly used mouse vascular tumor model. The results showed that hemangioendothelioma could be formed by subcutaneous injection of EOMA cells, which were rich in blood and could grow to a size suitable for HIFU ablation (**Figure 1**). The results of CEUS also showed that the blood inside the tumor was abundant, and the imaging features were consistent with the clinical manifestations of vascular tumors, showing fast advance and slow exit (**Figure 1F**). HE staining and CD31 fluorescence staining showed that the vascular lacuna-rich structure in the tumor was consistent with clinical vascular tumors (**Figures 1D, E**). The above results show that we have successfully established an animal model, which can be used in subsequent experiments.

In order to study whether pulsed ultrasound combined with microbubbles can change the abundant blood supply of vascular tumors and lead to the formation of thrombus improve and whether combined therapy can effectively ablate vascular tumors, we conducted contrast ultrasound, HE staining, cell apoptosis and other experiments. Our study found that pulsed ultrasound combined with microbubbles could cause thrombus to form inside the tumor. It was observed through CEUS that the enhanced effect of ultrasound inside the tumor was significantly weakened after the combined use of pulsed ultrasound and microbubbles, indicating the changes in internal hemodynamics and the existence of thrombosis. The results of Elisa also showed that the expression of D2D, FI, which is closely related to thrombosis in the pulsed ultrasound combined with microbubbles group was significantly increased (p < 0.001), and the initiation of exogenous clotting pathway FIII and endogenous clotting pathway FXII were significantly increased (p < 0.001) (**Figure 6**). It has been reported that thrombus formation can be restored by supplementing fibrinogen FI, which is a marker of clinical thrombosis (43). D-dimer is a global indicator of coagulation activation and fibrinolysis, and an indirect marker of thrombosis (44). FXII can maintain thrombus stability and locally regulate vascular permeability (45). The onset of blood clotting *in vivo* requires the accumulation of FIII tissue factor upstream of the developing thrombus and at the thrombo-vessel wall interface. Tissue factors have biological activity and are related to fibrin production within thrombus (46).

Through HE and TUNEL staining, we also found that the cells inside the tumor showed obvious apoptosis and necrosis after the combined treatment, and the results of CEUS also showed that coagulation necrosis was only observed in the PUCM+HIFU group (**Figure 5**). As shown in the **Figure 2A**, after the combination treatment, chromatin shrank and the nucleus was ruptured extensively. The results of apoptosis staining also showed that a large number of cells in the PUCM+HIFU underwent apoptosis and expressed the strongest green fluorescence (p < 0.001) (**Figure 2B**). It has been reported that the heat generated by HIFU can cause the temperature of exposed tissues to rise rapidly to more than 60 °C, and when the duration is more than 1 second, it will lead to immediate and irreversible cell death in most tissues (47). By regulating cell apoptosis and necrosis, new anti-tumor therapeutic strategies can be developed. For example, the combined use of the Orf virus and the PAK4 inhibitor exerts anti-tumor effects in breast cancer by inhibiting cell viability and inducing cell apoptosis (48); Curcumin reduces angiogenesis and induces apoptosis of tumor cells by inhibiting the expression of VEGF (49). In addition, targeting apoptosis inhibitors such as survivin can promote the apoptosis of tumor cells and improve the efficacy of chemotherapy (50).

The occurrence and development of vascular tumor is closely related to the expression of VEGF. Therefore, we studied the effects of different treatment methods on VEGF and its related signaling molecules. By immunofluorescence staining, we observed the weakest fluorescence signals of VEGF and CD31 in the combined treatment group (p < 0.001), indicating that the combined treatment can effectively lower the formation of blood vessels in the tumor. Elisa and qRT-PCR results also showed that the PUCM+HIFU group inhibited the expression of signaling molecules related to angiogenesis (**Figures 6**, **7**). Recent studies have found that VEGF mRNA expression in proliferative hemangiomas is significantly higher than that in regressive hemangiomas. VEGF mRNA expression was not found in the tissue of hemangioma at the regression stage and in the degenerated tissue. In conclusion, the decreased expression of VEGF is closely related to the disease outcome. A large number of studies have shown that inhibiting the secretion of VEGF in tumors can inhibit the growth of tumor blood vessels and play a role in regulating immunity and promoting therapy (51–53). HIFU can effectively inhibit tumor angiogenesis by directly destroying the tumor microvascular structure, reducing the expression of pro-angiogenic factors (such as VEGF, HIF-1α, HIF-2α), and combining with other therapeutic methods. After HIFU irradiation, the capillaries in the tumor tissue are destroyed, the arrangement of microvascular endothelial cells is disordered, and the vascular elasticity disappears (54). In addition, HIFU can also block the regenerated endothelial cells and the lumen they form, thereby blocking tumor vascular regeneration at multiple stages (55). Among patients with liver cancer, the expression levels of hypoxia-inducible factor-1 α (HIF-1α) and HIF-2α decreased significantly after HIFU treatment. These factors are important transcription factors that promote angiogenesis (56). Among breast cancer patients, the levels of VEGF significantly decreased after HIFU treatment, indicating that it has an inhibitory effect on angiogenic factors (57).

In conclusion, our study suggested that the treatment effect of HIFU could be improved by using pulsed ultrasound combined with microbubbles to form thrombus in hemangioendothelioma, after treatment, it can significantly promote the apoptosis of tumor cells and effectively inhibit the formation of blood vessels in the tumor. The results of this study provide further theoretical support for the clinical application of HIFU in the treatment of vascular tumors. However, due to the limitations of the experimental conditions, we were unable to conduct sufficient quantitative analyses regarding the changes in hemodynamics and thrombosis. We hope that in future research, we can further improve our study through methods such as *in vivo* microscopic imaging and non-invasive imaging.

## Data Availability

The original contributions presented in the study are included in the article/supplementary material. Further inquiries can be directed to the corresponding author.
